# Organism-Specific rRNA Capture System for Application in Next-Generation Sequencing

**DOI:** 10.1371/journal.pone.0074286

**Published:** 2013-09-20

**Authors:** Sai-Kam Li, Jun-Wei Zhou, Aldrin Kay-Yuen Yim, Alden King-Yung Leung, Stephen Kwok-Wing Tsui, Ting-Fung Chan, Terrence Chi-Kong Lau

**Affiliations:** 1 Department of Biology and Chemistry, City University of Hong Kong, Hong Kong SAR; 2 School of Biomedical Sciences, the Chinese University of Hong Kong, Hong Kong SAR; 3 School of Life Sciences, the Chinese University of Hong Kong, Hong Kong SAR; 4 State Key Laboratory of Agrobiotechnology, the Chinese University of Hong Kong, Hong Kong SAR; Université Libre de Bruxelles. BELGIQUE, Belgium

## Abstract

RNA-sequencing is a powerful tool in studying RNomics. However, the highly abundance of ribosomal RNAs (rRNA) and transfer RNA (tRNA) have predominated in the sequencing reads, thereby hindering the study of lowly expressed genes. Therefore, rRNA depletion prior to sequencing is often performed in order to preserve the subtle alteration in gene expression especially those at relatively low expression levels. One of the commercially available methods is to use DNA or RNA probes to hybridize to the target RNAs. However, there is always a concern with the non-specific binding and unintended removal of messenger RNA (mRNA) when the same set of probes is applied to different organisms. The degree of such unintended mRNA removal varies among organisms due to organism-specific genomic variation. We developed a computer-based method to design probes to deplete rRNA in an organism-specific manner. Based on the computation results, biotinylated-RNA-probes were produced by *in vitro* transcription and were used to perform rRNA depletion with subtractive hybridization. We demonstrated that the designed probes of 16S rRNAs and 23S rRNAs can efficiently remove rRNAs from *Mycobacterium smegmatis*. In comparison with a commercial subtractive hybridization-based rRNA removal kit, using organism-specific probes is better in preserving the RNA integrity and abundance. We believe the computer-based design approach can be used as a generic method in preparing RNA of any organisms for next-generation sequencing, particularly for the transcriptome analysis of microbes.

## Introduction

Prokaryotic RNomics has drawn unprecedented attention from molecular biologists and microbiologists in recent years. In studying RNomics, one would focus not only on the protein-coding mRNA, but also the small RNAs. A major obstacle in such studies is the relatively small amount of mRNA and small RNAs (sRNA) in comparison to the ribosomal RNA (rRNA), which constitutes over 90% of total prokaryotic RNA. In order to preserve and measure the subtle change in RNome, particularly the low abundant RNA, enrichment of mRNA or rRNA depletion is inevitable.

Various methods have been developed for eukaryotic system such as mRNA enrichment based on their polyadenylation status. However, this method is not ideal for prokaryotic mRNA purification as the poly(A) tails are associated with only 2-60% of the mRNA molecules [1]. Apart from that, the poly(A) tail of prokaryotic mRNA are generally shorter when compared to eukaryotic mRNA with adenosine ranging from 15 to 60, leading to a decreased binding efficiency [1]. Moreover, the poly(A) sequence in the prokaryotic mRNA are generally instable due to the short half-life [2]. Several other enrichment methods have been developed to increase the portion of RNA of interest such as the 5′-to-3′ exonuclease treatment of total RNA, in which only the 5’ monophosphate processed rRNA and tRNA are removed. Nevertheless, this approach can only remove 10-20% of rRNA from either Gram-positive or Gram-negative bacteria [3]. Another approach targets on a specific subset of RNAs which are associated with some proteins such as RNA chaperone Hfq [4,5]. Co-immunoprecipitation (Co-IP) of the protein-RNA complex can eliminate about half of rRNA and tRNA, according to Sittka’s report [4]. In addition to the above approaches, poly(A)-based rRNA depletion method was also used to enrich the prokaryotic mRNA in recent meta-transcriptomic study [6]. One of the most commonly used and efficient approaches is the probe-based subtractive hybridization rRNA capture. It uses probes correspond to either conserved sites on 23S or 16S bacterial rRNAs or rRNA operon [7,8]. As shown in several recent studies, target rRNA could be removed after hybridization with the probes or operons which are conjugated to magnetic beads for recovery [9,10,11,12,13]. In order to increase the probe-to-sample specificity, sample specific probes were also applied. Sample rRNA was amplified and appended with a T7 promoter, which then allows *in vitro* transcription of the probes [14].

In depleting rRNA, an unintended loss of mRNA is observed because of the off-target effect between the probes and the mRNA. Probe-based subtractive rRNAs capture approaches are proven to be better than exonuclease digestion in preserving RNA abundance and integrity [15]. The non-specific binding between subtractive probes and mRNAs varies among organisms such that variations within their transcriptomes may introduce different artifacts to the depletion processes. Therefore, we set out to develop an organism-specific rRNA depletion system which combines the computational and probe-based rRNA capture approaches. A computer program was developed for probe designing to ensure the uniqueness of the probe sequence in the genome and an efficient hybridization to capture the rRNA. In order to test our design, *Mycobacterium smegmatis*, a laboratory model for *Mycobacterium tuberculosis* was used [16]. The program was applied to design nine probes that may specifically bind to mycobacteria 16S or 23S rRNAs. By labeling the probe with biotinylated UTP, we showed that the probes could efficiently remove the mycobacterial rRNAs while maintaining the mRNA level.

## Materials and Methods

### Specific probe design for *Mycobacterium smegmatis* using OSPS

Probe selection was done by Organism-Specific Probe Selection (OSPS) which can be found with the user guide at https://sourceforge.net/projects/ospstools. The program is available in version 3.0 and version 3.0 LIR which have different system requirement. The version 3.0 LIR has an extra function which allows prediction of sites for self-annealing or forming secondary structures such as hair-pins. The program is written in JAVA with Biojava (19) and is divided into two modules - Pre-BLAST and Post-BLAST (Figure S1). Pre-BLAST module is responsible for generating the BLAST database (subject) and query sequences for the BLAST process while the Post-BLAST module is required for the design of specific probes. In this study, with the intake of two files – Genomic sequence (NC_008596.1) and Annotations (CP000480.1) of *Mycobacterium smegmatis* str. MC^2^155, all the coding sequences (CDS) were identified and extracted. The 23S (MSMEG_3756, MSMEG_4930) and 16S (MSMEG_3757, MSMEG_4931) ribosomal RNAs were identified, extracted and virtually fragmented to a window size of 100 bp (customable size) with leap size of 5 nucleotides (customable which must be >=1). The fragmented 16S and 23S rRNAs were then assigned as query and were subject to BLAST (18) against all the CDS. The results were then processed by the Post-BLAST module with an e-value less than 1 with both 90% of identity recovery and hit length. Final matches with smallest number of hits were then chosen for probe design.

### Culture of *Mycobacterium smegmatis* and total RNA extraction

*M. smegmatis* of strain MC^2^ 155 were grown in mycobacterium Middlebrook 7H9 medium (BBL) supplemented with 0.5% glycerol, 10% oleic acid-albumin-dextrose-catalase (OADC) (BBL) in a conical flask. The cultures were grown at 37°C with shaking of 100 rpm under subdue light. The culture was harvested at stationary phase when the optical density 600 nm (OD600) reached 2.0. Cells were collected from 50 ml culture. The cell pellets were lysed in 500 µl of 20 mg/ml lysozyme (Sigma-Aldrich) at 37°C for 30 min and then further lysed in 10 ml TRIzol reagent (Invitrogen) at room temperature for 10 min. The total RNA was then precipitated by isopropanol as instructed in TRIzol protocol. Since total RNA isolated for *M. smegmatis* usually contains some impurities, the isolated RNA was subject to a second-round purification using acidic phenol-chloroform (5:1, pH 4.5, Ambion). The purified RNA was reconstituted with 50 µl nuclease-free water. The RNA quantity and quality was analyzed on a NanoDrop ND-1000 spectrophotometer (Thermo, USA) and TBE agarose gel respectively.

### Reverse transcription PCR and probe cloning

First strand cDNA was synthesized using QuantiTect Reverse Transcription kit (QIAGEN) following the protocol provided by the manufacturer. One microgram of purified RNA in 12 µl of RNase-free water was mixed with 2 µl 7× gDNA Wipeout buffer. After incubation at 42°C for 2 min, 4 µl Quantiscript RT buffer (5×), 1 µl primer mix and 1 µl Quantiscript reverse transcriptase was added to the reaction and incubated at 42 °C for 30 min followed by 2 min incubation at 95 °C. OSPS-selected 16S or 23S sequences were amplified from the first strand cDNA by PCR using probe-specific primers incorporated with restriction sites. PCR reaction was performed with Phusion high-fidelity DNA polymerase (Finnzyme) with the following cycling profile: DNA denaturation at 94 °C for 3 min and 35 cycles of amplification at 94 °C for 30s, 60 °C for 30s, 72 °C for 1 min, plus a final extension step at 72 °C for 7 min. PCR products were then cloned into a modified pUC19 renamed as pT1 which carries a minimal SP6 promoter at the 3’ end of the multi-cloning site.

### Preparation of RNA probes by *in vitro* transcription

Probe-incorporated plasmid DNA was linearized with *Bam*HI (New England Biolabs) locating at the 5’ end of the cloned rRNA sequence. *In vitro* RNA transcription was done using MEGAscript^®^ SP6 kit (Ambion). The reaction mixture containing 1 µg of linearized plasmid DNA, 2 µl of each of 10 mM ATP, CTP, GTP, 0.5 µl of 10 mM UTP, 1.5 µl 10 mM Bio-11-UTP (Ambion) and 2 µl RNA polymerase was incubated at 37°C for 12 hours.

### Probe purification

The transcription product was mixed with Gel Loading Buffer II (Ambion) and separated on an 8% urea-acrylamide gel at constant current of 25 mA for 2 hours. The gel containing probe was visualized under the UV light and exercised. The exercised gel was transferred into 400 µl of RNA elution buffer (20 mM Tris-HCl pH 7.5, 250 mM sodium acetate, 1 mM EDTA pH 8.0, 0.25% SDS) followed by incubation at 42°C overnight. The probes were then purified with acidic phenol-chloroform (Ambion). Briefly, 400 µl of acidic phenol-chloroform (Ambion) was added to the gel-containing elution buffer. After vigorous sharking for 15 seconds and centrifugation at 12000 g for 15 min at 4 °C, the supernatants were collected and mixed with 40 µl of 5M ammonium acetate, 8 µl of GlycoBlue (Ambion) and 1.2 ml 96% ethanol. Probes were incubated at -20 °C for 2 hours and harvested by centrifugation at 12000 g for 15 min at 4°C. The probes were then washed with cold 70% ethanol and then dissolved in nuclease-free water.

### Ribosomal RNA capture by oligo hybridization

1 µg total RNA was mixed with 0.8 µg probe for 23S rRNA and 0.6 µg probe for 16S rRNA in 200 µl RSB-200 buffer (10 mM Tris-Cl pH 7.4, 3 mM MgCl_2_, 200 mM NaCl) containing 1.5 mM EDTA. The mixture was denatured at 70 °C for 10 min, then cooled down to 25 °C for probe-RNA hybridization. Meanwhile, Dynabeads M-280 Streptavidin (Invitrogen) was washed with 100 volumes of 200 µl RSB-300 (10 mM Tris-Cl pH 7.4, 3 mM MgCl_2_, 300 mM NaCl) containing 0.01% NP40 by centrifugation at 9000 g for 30 seconds. Then the samples were mixed with the streptavidin beads and incubated at 4 °C on a rotator for 15 min. After centrifugation at 9000 g for 30 seconds, the supernatant was transferred to a collection tube containing 40 µl of 5M ammonium acetate, 8 µl of GlycoBlue (Ambion) and 1.2 ml absolute ethanol. Another 200 µl RSB-200 supplemented with 1.5 mM EDTA was added to the RNA-probe-beads containing tube and incubated at 4°C for 5 min. The supernatant was transferred to the same collection tube after centrifugation at 9000 g for 30 seconds. The enriched RNA was precipitated by incubation at -20°C for 2 hours and harvested by centrifugation at 12000 g for 15 min at 4 °C. The purified RNA was dissolved in 20 µl of RNase free water. For comparison, the total RNA sample was also treated with the MICROB*Express*™ Bacterial mRNA Enrichment Kit (Ambion) following the manufacturer’s instructions.

### Denaturing agarose gel

Half of the enriched RNA sample was analyzed on 1.5% denaturing agarose gel (2.2 M formaldehyde in 1× 3-(N-morpholino) propanesulfonic acid/ MOPS electrophoresis buffer) with untreated *M. smegmatis* total RNA as control. The RNA was stained with SYBR® Safe (Life Technologies) and visualized under UV light.

### Validation of mRNA integrity by real-time PCR

Half of enriched RNA was used for reverse transcription reaction using QuantiTect Reverse Transcription kit (QIAGEN) as described previously. Real-time PCR was performed by using 2× Power SYBR Green PCR Master Mix (Applied Biosystems) in ABI 7500 Fast Real-time PCR system (Applied Biosystems). The PCR cycling program initiated from denaturation at 95 °C for 10 min followed by 40 cycles at 95 °C for 15 s and at 60 °C for 1 min. Melting curves were also determined by an auto-dissociation program. The house-keeping gene MSMEG_5072 was measured for normalization. Comparative C_T_ method (2^-ΔΔCT^) was used to calculate the relative expression level of the target 16S rRNA, 23S rRNA and other mRNA.

## Results

### Removal of rRNA using selected probes

**Figure 1:**
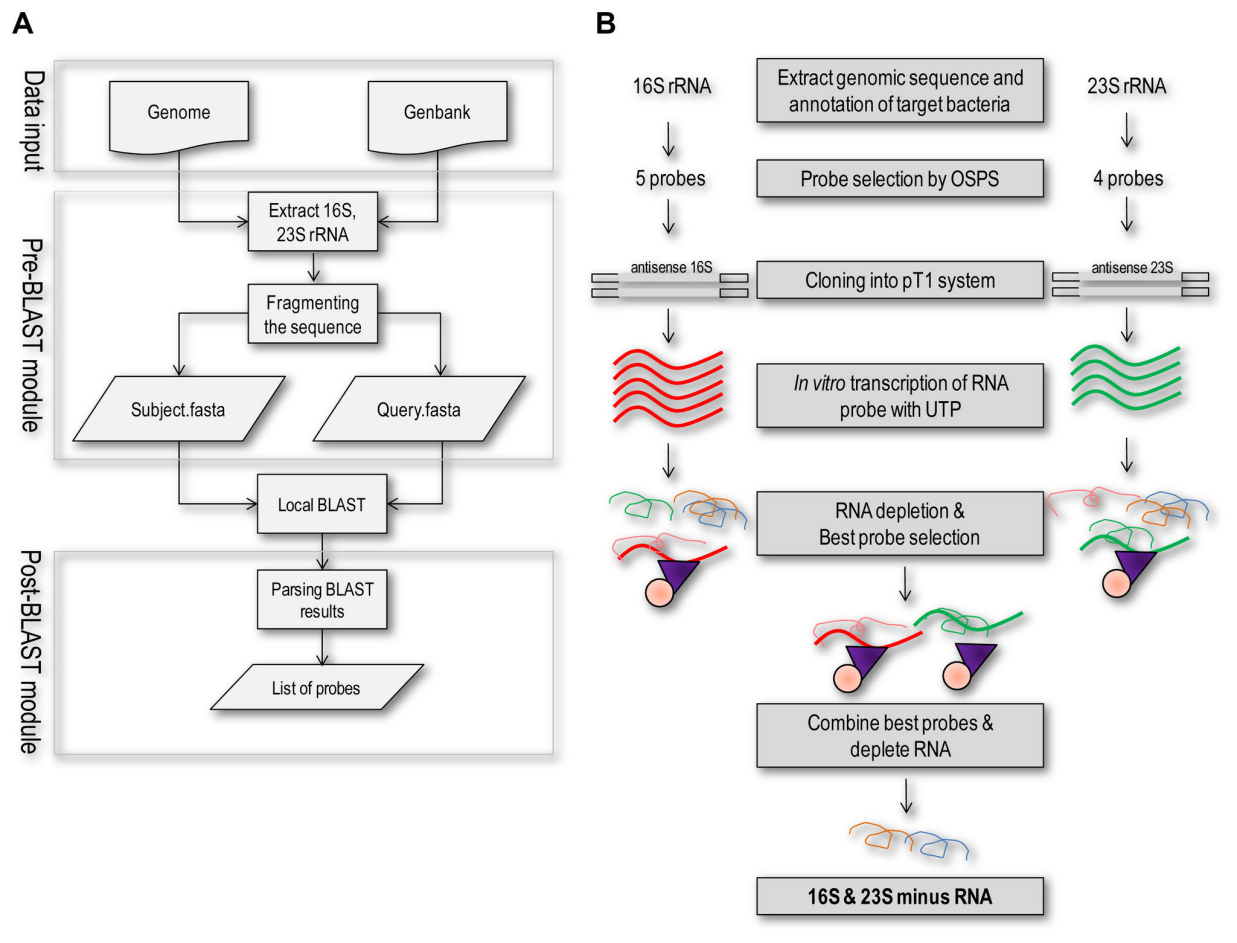
Workflow illustrating the procedures for Organism-Specific Probe Selection and rRNA depletion. **A**. Workflow of Organism-Specific Probe Selection (OSPS) program. OSPS was used to screen for unique sequences of 16S rRNAs and 23S rRNAs that have no significant similarity to other transcripts in the same organism. **B**. Overall procedures of rRNA depletion. Sequences for probes were amplified and cloned into an in-house pT1 system, and the RNA probes were which by *in* *vitro* transcribed with biotinylated UTP and tested for rRNA depletion efficiency. The best probes were selected and combined for further rRNA depletion.

**Figure 2:**
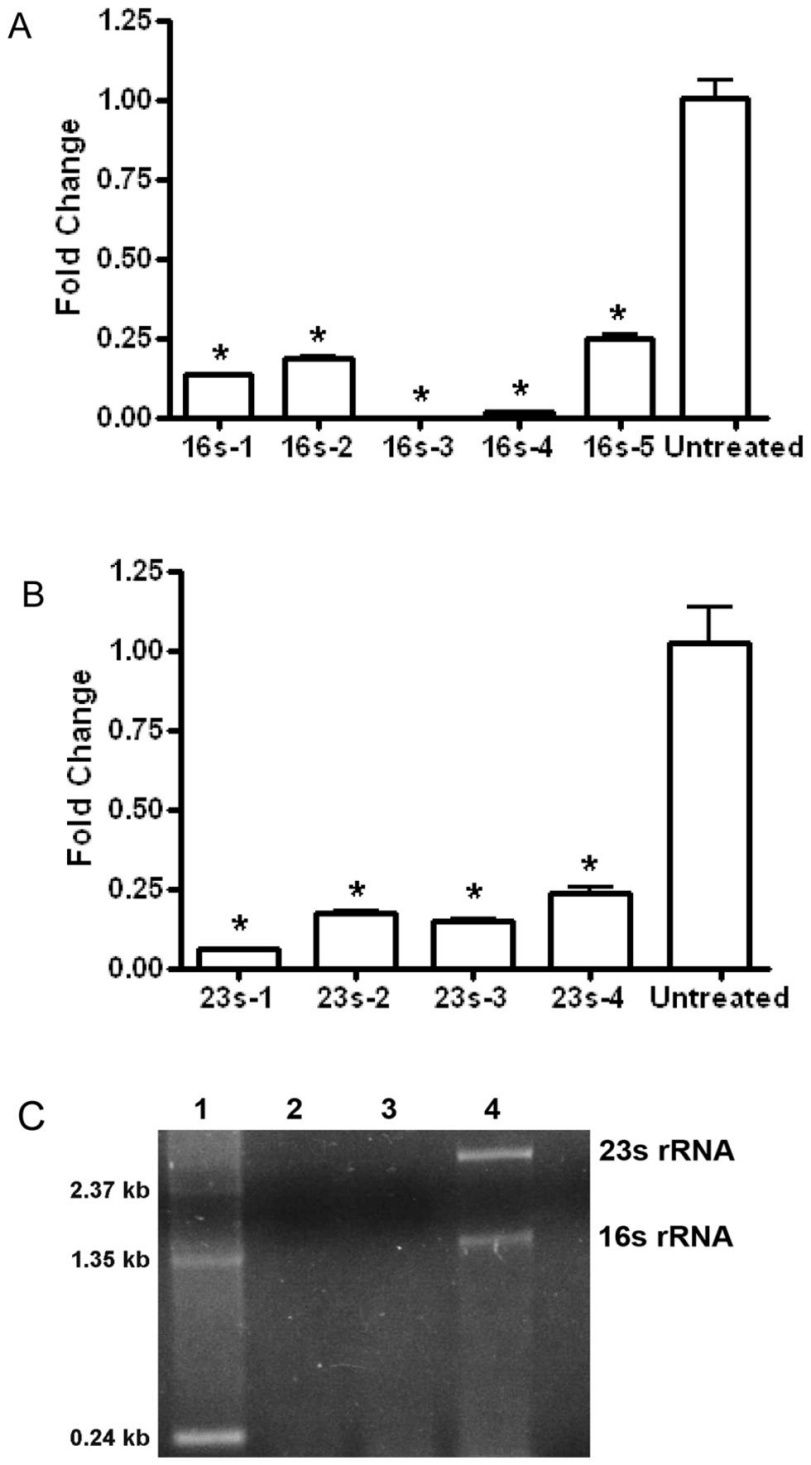
Evaluation of the removal efficiency of rRNA from total RNA using probes designed by OSPS. The rRNA levels were measured before and after rRNA depletion by real-time PCR. Untreated sample was included for comparison and MSMEG_5072 gene was used as a normalization gene. * means p-value <0.001. **A**. Depletion efficiency for 16S rRNA. Five probes of 16S rRNAs were used for depletion separately. **B**. Depletion efficiency for 23S rRNA. Four probes of 23S rRNAs were used to deplete 23S rRNA. **C**. Depletion of rRNAs from total RNA of *Mycobacterium smegmatis* by combined probes. Total RNA before and after depletion were loaded and separated on an agarose gel. Lane 1, 1.5 µg RNA marker; lane 2, RNA sample was depleted using the MICROB*Express*™ Bacterial mRNA Enrichment Kit (Ambion); lane 3, RNA sample was depleted by combined probes (16S-3 and 23S-1); lane 4, 0.5 µg of untreated RNA.

The workflow of the program Organism-Specific Probe Selection (OSPS) and the experimental procedures of rRNA depletion were illustrated in Figure 1A and 1B respectively. Various probes which do not hit any transcripts in the current nucleotide database of *Mycobacterium smegmatis* were selected by OSPS for 16S and 23S rRNAs respectively (Table 1), and were *in vitro* transcribed with biotinylated UTP. The biotinylated probes were then purified and used for rRNA depletion of total RNA extracted from *Mycobacterium smegmatis*. The depletion efficiency of each single probe was determined by real-time PCR. As indicated in Figure 2A and 2B, all the designed probes for 16S (16S-1, 16S-2, 16S-3, 16S-4 AND 16S-5) and 23S probes (23S-1, 23S-2, 23S-3 and 23S-4) significantly removed the rRNAs (more than 75% removal of rRNA). Moreover, in this experiment, probe 16S-3 and 23S-1 were considered as the most efficient probes to remove 16S rRNAs and 23S rRNAs respectively from total RNA. These two probes were then combined to deplete 16S and 23S rRNAs simultaneously and compared with the rRNA depletion results from using MICROB*Express*™ Bacterial mRNA Enrichment Kit (Ambion). In Figure 2C, Lane 4 showed the two typical bands of 23S and 16S rRNA on an agarose gel. rRNA-depleted RNAs prepared by using MICROB*Express*™ Bacterial mRNA Enrichment Kit (Ambion) or using our probes were shown in lane 2 and lane 3 respectively. Both the commercial kit and our designed oligos efficiently removed the 23S and 16S rRNA to a level that was invisible on gel.

**Table 1:** Probes of rRNAs selected by Organism-Specific Probe Design (OSPS).

Probe Name	Target sequence	Length	Number of Hit (E-value <1)
16S-1	gtagccggcctgagagggtgaccggccacactgggactgagatacggcccagactcctacgggaggcagcagtggggaatattgcacaatgggcgcaagc	100	0
16S-2	gacgccgcgtgagggatgacggccttcgggttgtaaacctctttcagcacagacgaagcgcaagtgacggtatgtgcagaagaaggaccggccaactacg	100	0
16S-3	tgccagcagccgcggtaatacgtagggtccgagcgttgtccggaattactgggcgtaaagagctcgtaggtggtttgtcgcgttgttcgtgaaaactcac	100	0
16S-4	agcttaactgtgggcgtgcgggcgatacgggcagactagagtactgcaggggagactggaattcctggtgtagcggtggaatgcgcagatatcaggagga	100	0
16S-5	ggtggcgaaggcgggtctctgggcagtaactgacgctgaggagcgaaagcgtggggagcgaacaggattagataccctggtagtccacgccgtaaacggt	100	0
23S-1	atctcagtacccgtaggaagagaaaacaaaatgtgattccgtgagtagtggcgagcgaaagcggaggatggctaaaccgtatgcatgtgataccgggtag	100	0
23S-2	gggttgtgtgtgcggggttgtgggacctatctttccggctctacctggctggagggcagtgagaaaatgttgtggttagcggaaatggcttgggatggcc	100	0
23S-3	aggccaatcaaactccgtgatagctggttctccccgaaatgcatttaggtgcagcgtcgcatgtttcttgccggaggtagagctactggatggccgatgg	100	0
23S-4	cgaaagtcgggactagtgatccggcacctctgagtggaaggggtgtcgctcaacggataaaaggtaccccggggataacaggctgatcttccccaagagt	100	0

### Evaluation of the integrity of mRNA

**Figure 3:**
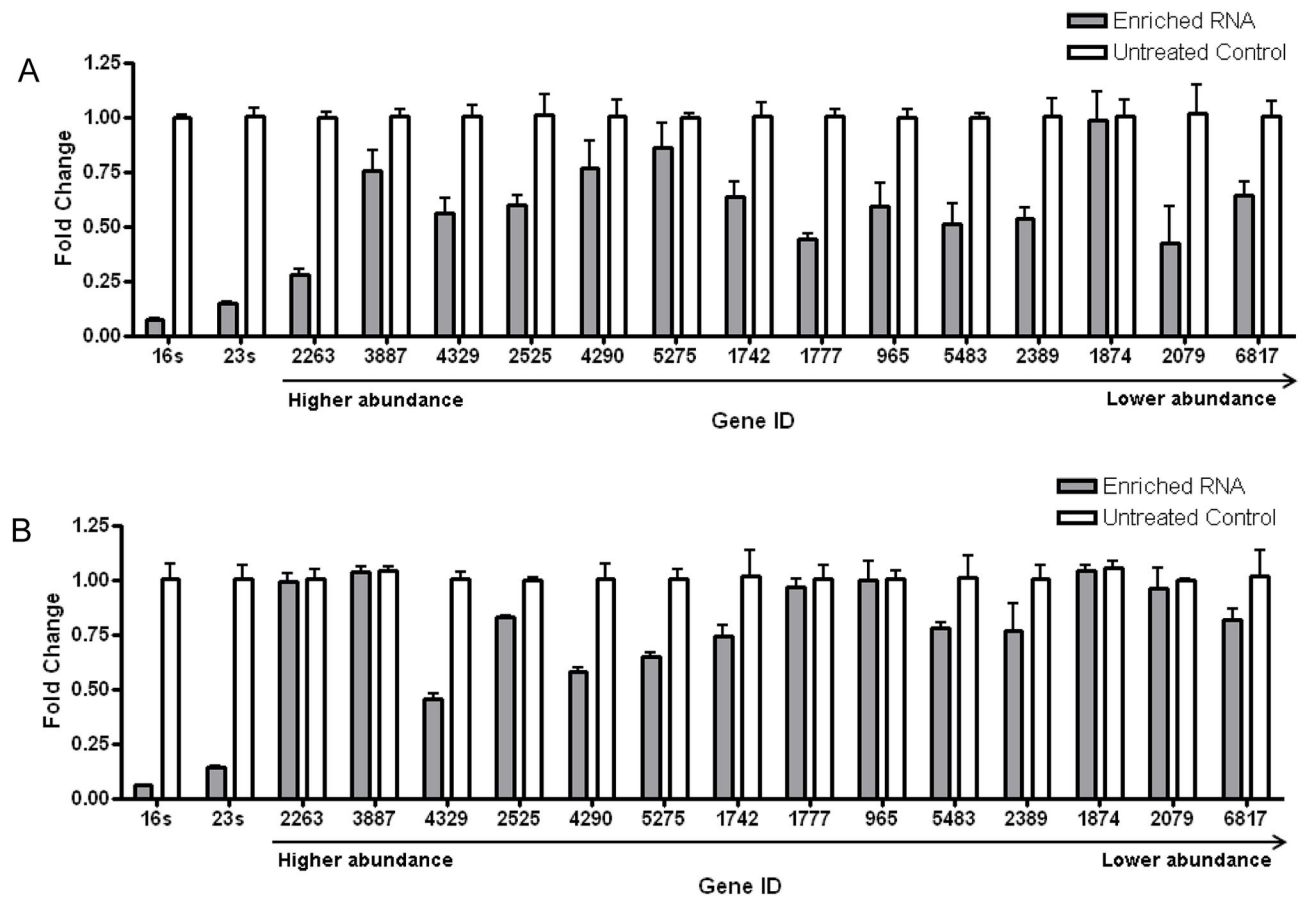
Integrity of mRNA after rRNA depletion was determined by real-time PCR. The expression level of rRNA and 14 genes of different abundance were measured before and after rRNA depletion using MSMEG_5072 gene as a housekeeping control. Abundance of selected genes and the gene ID were shown. **A**. Relative fold change of selected mRNA and rRNA after depletion using MICROB*Express*™ Bacterial mRNA Enrichment. **B**. Fold change of selected mRNA and rRNA after depletion using probes designed by OSPS.

As shown in Figure 3, both MICROB*Express*™ Bacterial mRNA Enrichment Kit (Ambion) and our OSPS probes reduced the amount of 16S and 23S rRNAs to a level lower than 0.25 fold relative to the original amount of rRNAs. In order to evaluate the effect on the rRNA depletion probes on the integrity of mRNA, the transcription level of several selected genes was determined by real-time PCR in parallel. Fourteen genes ranging from high abundance to low abundance in *M. smegmatis* were studied and compared to the level in untreated RNA sample. Compared to our OSPS probes, using MICROB*Express* ^TM^ caused an overall greater decrease in transcript amounts for genes of different abundances (Figure 3). For instance, MICROB*Express* ^TM^ caused a decrease in transcript level by 50-75% (Figure 3A) for highly abundant genes such as MSMEG_2263 (*hybC*) and MSMEG_2525 (amino acid permease); moderately abundant genes such as MSMEG_1742 (oxidoreductase), MSMEG_1777 (*UsfY*), and MSMEG_0965 (porin); and lowly abundant genes MSEMG_2389 (*hup*) and MSMEG_2079 (alcohol dehydrogenase). In comparison, OSPS retained 100% transcript level for MSMEG_2263, MSMEG_1777, MSMEG_0965, and MSMEG_2079. OSPS also performed better in retaining the transcript levels of MSMEG_2525 and MSMEG_2389 (Figure 3B). However, both methods caused a significant transcript level reduction in MSMEG_4329 (propionyl-CoA carboxylase subunit beta) and MSMEG_4290 (*glnA*).

## Discussion

Ribosomal RNA contamination has been a problem in studies that focus on either coding or non-coding RNAs. In account of the sequence complexity of bacteria, probes that are designed based on the conserved sequences of bacteria rRNAs may also bind to and hence deplete some coding RNAs as illustrated in this study. Here we propose the idea of using organism-specific probes instead of using universal probes that target conserved rRNA sequences in bacteria. We demonstrated our organism-specific rRNA depletion method on *Mycobacterium smegmatis*. This method consists of three stages. Firstly, Organism-Specific Probe Selection (OSPS) program was used to screen for probes that are specific to mycobacterial rRNAs and have no significant similarity to other mycobacterial transcripts. Secondly, probes were designed and produced using an in-house plasmid system in combination with easily available commercial kits and reagents. Thirdly, the generated probes were applied to deplete the target RNA from total RNA.

We compared our in-house organism-specific method with the commercially available MICROB*Express* ^TM^ kit which is a bacteria-specific subtractive hybridization rRNA removal kit. Our results demonstrate that computationally-selected probes can efficiently deplete 23S and 16S rRNAs from total RNA in *M. smegmatis* to a level comparable to the commercial kit. In spite of no similarity between ours probes and mRNA in *M. smegmatis*, off-target mRNA depletion was still observed. This can be due to the nonspecific hybridization of the DNA probes to the mRNA as some bases on the mismatch DNA probe can effectively forms Watson-Crick pairings in the nonspecific duplexes [17]. Yet when compared to the commercial kit, using our probes ensures a better mRNA integrity for genes of various abundance. RNA samples prepared by this method, when assayed for expression levels either by microarrays or deep-sequencing, will give a more robust analysis of the transcriptome.

Our findings also suggest a potential system for bacteria-specific rRNA depletion using streptavidin beads. In theory, this program could be applied to any organisms. However, we anticipate a various efficiency of rRNA depletion depending on the genome complexity of particular organisms. This method can also be further advanced by modifying the program and the experimental procedures to suit different purposes. For example, the program may be modified to remove RNA species other than 23S and 16S rRNAs by selecting the specific sequence on the RNA of interest. In this study, biotinylated-UTP was incorporated into the RNA probes which are then able to bind to streptavidin beads. In consideration of the cost of biotinylated UTP, an alternate cost-effective probe “labeling” methods can be applied by using RNA affinity tags. For instance, streptavidin aptamers could be cloned in front of or at the end of the probe sequence [18]. By *in vitro* transcription, the RNA probe will be linked with an affinity tags which can be immobilized onto streptavidin beads. Target RNA species such as rRNAs will then be bound onto the streptavidin and therefore be removed.

In summary, the combination of OSPS and our in-house pT1 system should provide a tool for depleting ribosomal RNA in an organism-specific manner. We anticipate this strategy to be widely utilized in preparing RNA samples extracted from diverse organisms for application in Next-generation sequencing.

### Computer program

The computer program and manual described in this paper is available at https://sourceforge.net/projects/ospstools.

## Supporting Information

Figure S1 **Designing probes using Organism-Specific Probe Selection (OSPS) program.**
(DOCX)
