# Supplementary material for: Organism-Specific rRNA Capture System for Application in Next-Generation Sequencing
Source: PLoS One. 2013 Sep 20;8(9):e74286. doi: 10.1371/journal.pone.0074286 (PMC3779251; doi:10.1371/journal.pone.0074286)
Supplement: Figure S1 — Designing probes using Organism-Specific Probe Selection (OSPS) program. (DOCX) [file pone.0074286.s001.docx]

**
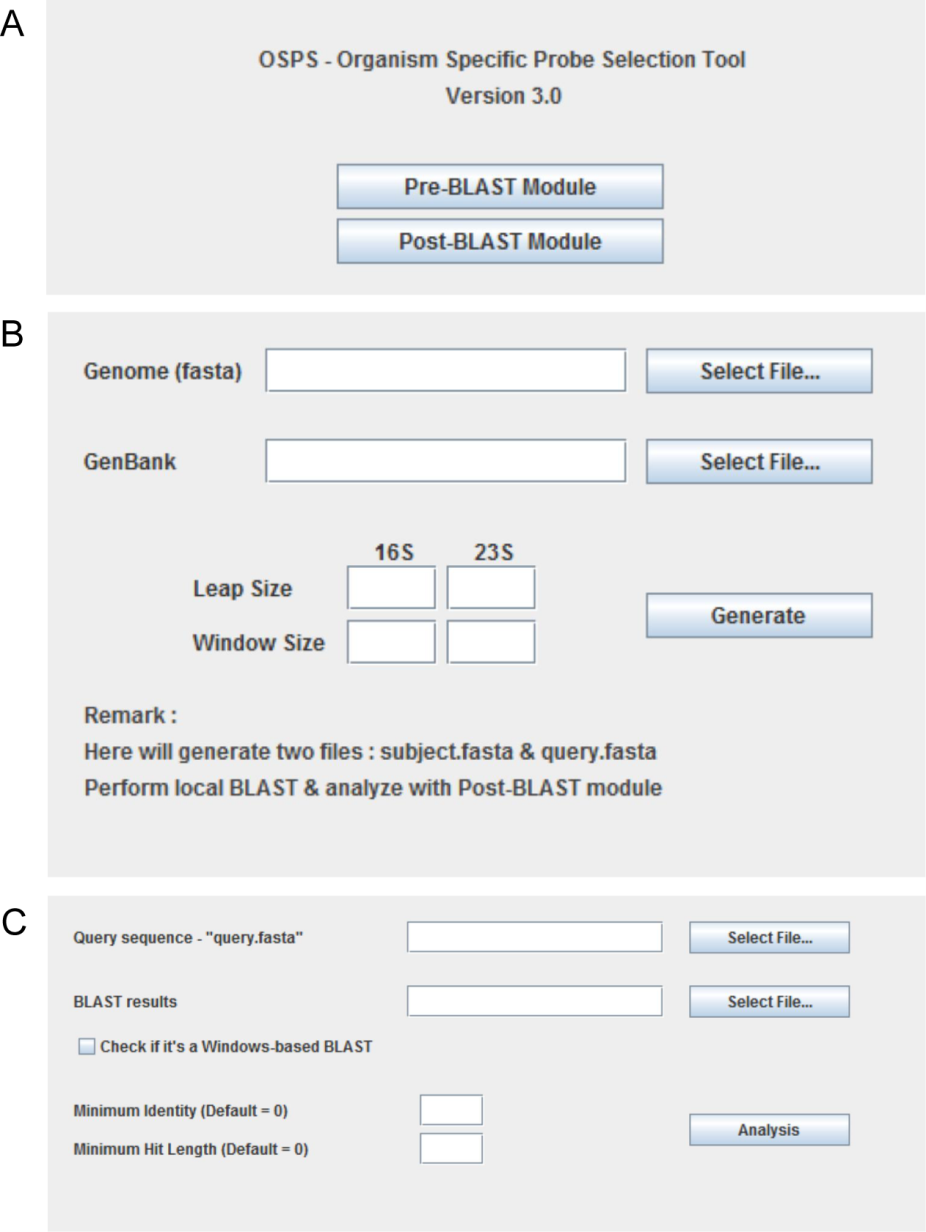
**

**Figure S1. Designing probes using Organism-Specific Probe Selection (OSPS) program. A.** OSPS is divided into two modules including the Pre-BLAST module and Post-BLAST module. **B.** Pre-BLAST module is responsible for generating the Database (Subject) and Query sequences for its subsequent process. **C.** In Post-BLAST module, the fragmented 16S and 23S rRNAs were then assigned to query and were then subjected to BLAST against the coding sequences. A list of sequences for probe design will be generated from the Post-BLAST module.
